# 4-Methylumbelliferone inhibits ovarian cancer growth by suppressing thymidine phosphorylase expression

**DOI:** 10.1186/s13048-014-0094-2

**Published:** 2014-10-11

**Authors:** Ryosuke Tamura, Yoshihito Yokoyama, Hidemi Yoshida, Tadaatsu Imaizumi, Hideki Mizunuma

**Affiliations:** Department of Obstetrics and Gynecology, Hirosaki University Graduate School of Medicine, 5 Zaifu-cho, Hirosaki, 036-8562 Japan; Department of Vascular Biology, Institute of Brain Science, Hirosaki University Graduate School of Medicine, 5 Zaifu-cho, Hirosaki, 036-8562 Japan

**Keywords:** 4-Methylumbelliferone, Ovarian cancer, Peritonitis carcinomatosa, Hymidine phosphorylase, HRA cells

## Abstract

**Background:**

4-Methylumbelliferone (4-MU), a hyaluronan (HA) synthesis inhibitor, has antitumor activity in cancer cells. However, few studies have focused on its effects on ovarian cancer. The aim of this study was to investigate the effects of 4-MU on ovarian cancer and to elucidate its mechanism of action.

**Methods:**

The HRA human ovarian serous adenocarcinoma cell line was used in this study. The effects of 4-MU on cell proliferation, migration, and invasion were determined by using *in vitro* assays as well as an *in vivo* rat peritoneal carcinomatosis model. The expression of HA synthase (HAS), CD44 HA receptor, vascular endothelial growth factor (VEGF), and thymidine phosphorylase (TP) mRNA in HRA cells was analyzed by quantitative reverse transcriptase-polymerase chain reaction (qRT-PCR).

**Results:**

4-MU administration inhibited the growth of peritoneal tumors and significantly prolonged survival. *In vitro* experiments showed that 4-MU inhibited HRA cell proliferation in a dose-dependent manner, while it did not affect HRA cell invasion and migration. 4-MU significantly decreased TP mRNA expression in HRA cells. On the other hand, since HAS2, CD44, and VEGF endogenous mRNA expression levels were very low in HRA cells, it was impossible to evaluate the effect of 4-MU treatment.

**Conclusions:**

These results suggest that 4-MU exerts its antitumor effect on ovarian cancer through suppressing TP expression.

## Background

Epithelial ovarian cancer is the most lethal malignancy in women because it is usually diagnosed at the severe peritonitis carcinomatosa advanced stage of the disease. Epithelial ovarian cancer is the sixth and eighth leading cause of cancer mortality among women in developed and developing countries [1]. The worldwide estimated number of new ovarian cancer cases increased from 137,600 in 1980 to 225,000 in 2008 [1]. There are different strategies for treating advanced ovarian cancer. Cytoreductive surgery followed by adjuvant chemotherapy is commonly recommended as the primary treatment for advanced (stage III/IV) epithelial ovarian cancer. Alternatively, neo-adjuvant chemotherapy may be selected for patients with bulky stage III/IV tumors for which surgery would be suboptimal. Postoperatively, the combination of a taxane and carboplatin is used as first-line chemotherapy [2]. Moreover, taxanes, carboplatin, cisplatin, liposomal doxorubicin, gemcitabine, etoposide, or topotecanin in combination or as single agents are acceptable as second- or third-line therapies for relapsed disease. Thus, chemotherapy plays an important role in ovarian cancer treatment. However, some patients with advanced ovarian cancer develop chemoresistance, which makes it difficult to prevent the development of peritonitis carcinomatosa, leading to reduced quality of life and a poor prognosis. Since the effects of the available treatments for ovarian cancer are restricted, it is necessary to develop new drugs.

Recent clinical trials showed that targeted drugs, such as bevacizumab [3], poly(adenosine diphosphate-ribose) polymerase inhibitors [4], farletuzumab [5], and trabectedin [6], are effective for ovarian cancer treatment and some of them are used clinically. However, because of concerns, including adverse reactions and cost-effectiveness, the clinical advantages of targeted drugs for ovarian cancer treatment remain unclear.

Hyaluronan (HA) is a high-molecular-weight glycosaminoglycan and a major component of the pericellular matrix. HA, which is synthesized by hyaluronan synthase (HAS), regulates multiple cellular functions [7]. The cellular effects of HA are mediated through the CD44 HA receptor [8]. CD44 has also been recently recognized as a cancer stem cell surface marker in several cancer types [9,10], and CD44 expression in cancer cells promoted bone metastasis by enhancing tumorigenicity, cell migration and invasion, and HA production [11]. 4-Methylumbelliferone (4-MU) is an HA synthesis inhibitor [7]. 4-MU blocks HA synthesis by inhibiting glucuronidation by endogenous glucuronosyltransferase, which results in depletion of uridine diphosphate glucuronic acid [7]. 4-MU also downregulates HAS2 and HAS3 expression [12]. Recent studies showed that 4-MU had an antitumor effect on prostate, breast, and hepatocellular carcinomas through the inhibition of HA synthesis [13-15]. Therefore, the CD44-HA interaction may be a promising target for therapeutic intervention of metastases. However, the clinical implications of 4-MU administration and ovarian cancer growth have not been investigated.

This study was conducted to investigate the antitumor potential of 4-MU against human ovarian cancer cells and to elucidate its mechanism of action.

## Methods

### Chemicals and cell culture

4-MU was purchased from Tokyo Chemical Industry (Tokyo, Japan). HRA cells, which were derived from a human ovarian serous adenocarcinoma [16], were kindly provided by Dr. Y. Kikuchi (National Defense Medical College, Japan) and were maintained in RPMI 1640 medium (Invitrogen, Tokyo, Japan) supplemented with 10% fetal bovine serum (FBS), 100 U/ml penicillin, and 100 μg/ml streptomycin at 37°C in a humidified atmosphere with 5% CO_2_/95% air. This cell line was authenticated as being ovarian in origin with a written guarantee. 4-MU stock solution for *in vitro* experiments was dissolved in medium containing 1% dimethylsulfoxide (DMSO) (Sigma-Aldrich, St Louis, MO) and the final concentration of DMSO in the medium was adjusted to 0.1%.

### Ethics statement

Animal experiments were approved by the Animal Research Committee of Hirosaki University (M13024) and all animals were cared for and handled in accordance to the Rules for Animal Experimentation of Hirosaki University and animal practices as defined by the national and local animal welfare bodies (Guide for the Care and Use of Laboratory Animals published by the National Institutes of Health). Animals were sacrificed according to the Guidelines for Euthanasia of Rodents using carbon dioxide.

### Peritoneal carcinomatosis model

Eight-week-old female F344/NJcl-rnu/rnu nude rats (CLEA Japan, Inc., Tokyo, Japan) were used in this study. Rats were group-housed in plastic cages with stainless-steel grid tops under a 12-hour light/dark cycle and given free access to water and food. Laparotomy was performed under sterile conditions and with general anesthesia using pentobarbital. Anesthesia was maintained up to 30 minutes with good analgesia and muscle relaxation. The omentum was accessed via a 4-cm midline incision in the abdomen and subsequently extirpated by ligating the gastroepiploic vessels and hilum of the spleen [17]. After hemostasis, the abdominal wall was closed in 2 layers by using 3/0 polyglactin (Vicryl®, Ethicon, Tokyo, Japan). No animals died as a result of the operative procedure. On the second day after surgery, HRA cells (3.7 × 10^7^ cells) were inoculated into the peritoneal cavity of the rats by injecting cells intraperitoneally with a 22-gauge needle. The rats were divided into 2 groups (n = 5 each). In the 4-MU-treated group, rats were administered 4-MU (100 mg per body) dissolved in 2.0 ml of 0.4% carboxymethylcellulose (CMC) solution (Sigma-Aldrich) intraperitoneally daily starting on the day when the cells were inoculated. The same volume of 0.4% CMC solution without 4-MU was administered intrapetitoneally to the rats in the control group. We allowed a subset of animals from each group to survive until the humane endpoints defined by inability to access food or water or increased effort due to progressing abdominal ascites. Rats were sacrificed by carbon oxide asphyxiation at the defined humane endpoints. Their health conditions were observed daily and all efforts were made to minimize suffering.

### Cell proliferation assay

Cell proliferation was assayed by using a Cell Counting Kit-8 (CCK-8; Dojin Laboratories, Kumamoto, Japan). HRA cells were cultured overnight in 96-well microplates at 2 × 10^3^ cells per well with 100 μl of medium. Further, the cells were treated with 0, 0.2, 0.6, and 1.0 mM 4-MU and cultured for 72 hours. Cell viability was assessed 3 hours after the addition of CCK-8 by measuring A_450_ with a Multiskan FC microplate reader (Thermo scientific, Yokohama, Japan). A preliminary study using this kit showed that absorbance was directly proportional to the number of cells. The experiment was conducted 5 times.

### Cell invasion and migration assay

Cell invasion and migration were assessed with a Cytoselect Cell Invasion Assay (Cell Biolabs, Inc., San Diego, CA) and a Cytoselect Cell Migration Assay (Cell Biolabs, Inc.), respectively, according to the manufacturer’s protocol. For invasion assays, HRA cells (1.0 × 10^5^ cells per well) in serum-free medium containing 0, 0.2, 0.6, or 1.0 mM 4-MU were placed in the upper chamber, which had an 8.0-μm pore size membrane coated with a uniform layer of basement membrane matrix solution, and medium with 10% FBS was placed in the lower chamber. After 24 hours, the cells from the underside of the membrane were removed by tilting the membrane chamber in Cell Detachment Solution. Lysis Buffer/CyQUANT GR dye solution was added to each well and the fluorescence of the mixture was measured by using a fluorescence plate reader (Fluoroskan Ascent, Thermo Scientific) at excitation and emission wavelengths of 480 nm and 520 nm, respectively. For migration assays, HRA cells (1.0 × 10^5^ cells per well) in serum-free medium containing 0, 0.2, 0.6, or 1.0 mM 4-MU were placed in the upper chamber, which had an 8.0-μm pore size membrane without basement membrane matrix solution, and medium with 10% FBS was placed in the lower chamber. After 3 hours, the cells that had migrated to the lower surface were quantified by using CyQUANT GR dye as described above. Each experiment was performed in triplicate.

### Real-time quantitative polymerase chain reaction (qPCR)

Expression levels of HAS2, HAS3, CD44, vascular endothelial growth factor (VEGF), and thymidine phosphorylase (TP) were determined in HRA cells cultured with or without 1.0 mM 4-MU for 24 hours. Total RNA was extracted from the cells by using an Illustra RNAspin Mini RNA Isolation Kit (GE Healthcare, Piscataway, NJ) and cDNA was synthesized with an iScript Advanced cDNA Synthesis Kit (Bio-Rad, Hercules, CA). Real-time qPCR was performed by using a CFX96 real-time PCR detection system (Bio-Rad) and a SsoAdvanced SYBR Green Supermix solution (Bio-Rad), according to the manufacturer’s specifications. The amplification conditions were as follows: 30 seconds at 95°C, followed by 95°C for 5 seconds and 60°C for 30 seconds for 40 consecutive cycles. After amplification, a melting curve from 65°C to 95°C at 0.5°C increments and 5 seconds per step was generated with continuous monitoring of fluorescence. The melting curves and quantitative analysis of the data were performed by using CFX manager Version 2.1 software (Bio-Rad). The mRNA levels of HAS2, HAS3, CD44, TP, and VEGF in a sample were normalized to the amount of 18S rRNA [18]. The sequences of the primers were as follows:

HAS2 forward, 5′-CAGCCTCATCTGTGGAGATGGTAA-3′

HAS2 reverse, 5′ -CCAGAGGTCCACTAATGCACTGAA-3′

HAS3 forward, 5′ -TGCGACTCTGACACTGTGCTG-3′

HAS3 reverse, 5′ -GGAAATCCATGAGTCGTACTTGTTG-3′

CD44 forward, 5′ -CTCCGGACACCATGGACAA-3′

CD44 reverse, 5′-CCACGTGGAATACACCTGCAA-3′

TP forward, 5′-GGCTGCTGTATCGTGGGTCA-3′

TP reverse, 5′ -GAACTTAACGTCCACCACCAGAG-3′

VEGF forward, 5′-TGGAGTGTGTGCCCACTGAG-3′

VEGF reverse, 5′- TGCATTCACATTTGTTGTGCTGTAG-3′

18S rRNA forward, 5′-ACTCAACACGGGAAACCTCA-3′

18S rRNA reverse, 5′ -AACCAGACAAATCGCTCCAC-3′

Specific primer sets for HAS2, HAS3, CD44, VEGF, and TP were purchased from Takara Bio, Inc. (Otsu, Japan). 18S rRNA specific primers were purchased from FASMAC (Atsugi, Japan).

### Western blot analysis

Cell lysates (50 μg protein) were prepared from cultured HRA cells treated with 0, 0.2, 0.6, and 1.0 mM 4-MU for 72 hours, electrophoresed through a 12.5% sodium dodecyl sulfate polyacrylamide gel, and blotted as described previously [19]. The protein concentration was determined using Bradford’s method. The blots were probed with the following diluted antibodies for 2 hours: TP (Proteintech, Chicago, IL) (Catalog number: 12383-1-AP) at 1:1000 and β-actin (Sigma-Aldrich) at 1:2000. The membranes were then incubated for 1 hour with the appropriate biotinylated secondary antibodies, transferred to avidin-biotin-peroxidase complex reagent, and incubated in this solution for 30 minutes. Diaminobenzidine was used as a substrate.

### Statistical analysis

Survival rates were calculated by using the Kaplan–Meier method and the statistical significance of differences in the cumulative survival curves between the groups was evaluated using the Wilcoxon test. Other statistical analyses were carried out with Student’s *t*-test. *P* values < 0.05 were considered statistically significant.

## Results

### Anti-tumor effect of 4-MU in a peritonitis carcinomatosa model

**Figure 1:**
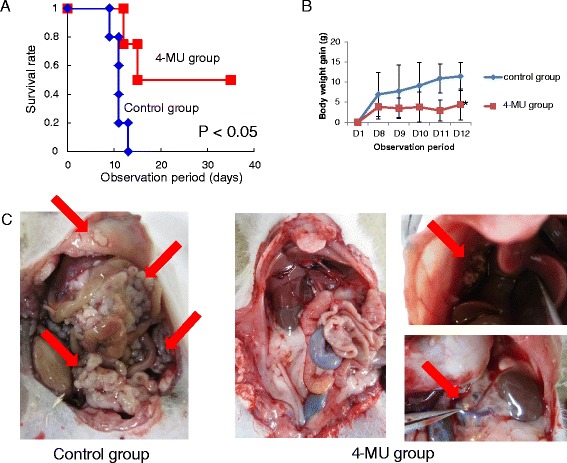
**Anti-tumor effect of 4-MU in a peritonitis carcinomatosa model. (A)** Rats in the control group did not survive after day 13. The survival times were significantly prolonged in the 4-MU-treated group compared with the control group (*P* < 0.05). **(B)** There was a significant difference in body weight gain between the 2 groups due to increased malignant ascites. *P < 0.05 **(C)** Differences in peritoneal tumors between the 2 groups. Rats in the control group developed large peritoneal tumors, whereas tumor sizes were smaller in rats in the 4-MU-treated group. The arrows indicate peritoneal tumors.

The survival periods of the control and 4-MU-treated groups were compared. All rats in the control group died by day 13. 4-MU administration in the 4-MU-treated group was discontinued after day 14 and survival was monitored thereafter for another 3 weeks. The survival times were significantly longer in the 4-MU-treated group than in the control group (*P* < 0.05, Figure 1A). Hemorrhagic ascites developed in the control group, whereas they were absent or mild in the 4-MU-treated group. Therefore, there was significant difference in body weight gain between the 2 groups (Figure 1B). As shown in Figure 1C, peritoneal tumors developed in the control group. Two rats in the 4-MU-treated group survived for 5 weeks and were sacrificed with carbon dioxide as described in Ethics statement section, although they reached the defined humane endpoints at the experimental endpoint. The post-mortem examination showed that their intraperitoneal tumors were much smaller than those in the control rats even though 4-MU administration was discontinued after day 14 (Figure 1C). Neither pleural effusion nor pleural metastasis was observed in both groups.

### Effects of 4-MU on cell proliferation, invasion, and migration

**Figure 2:**
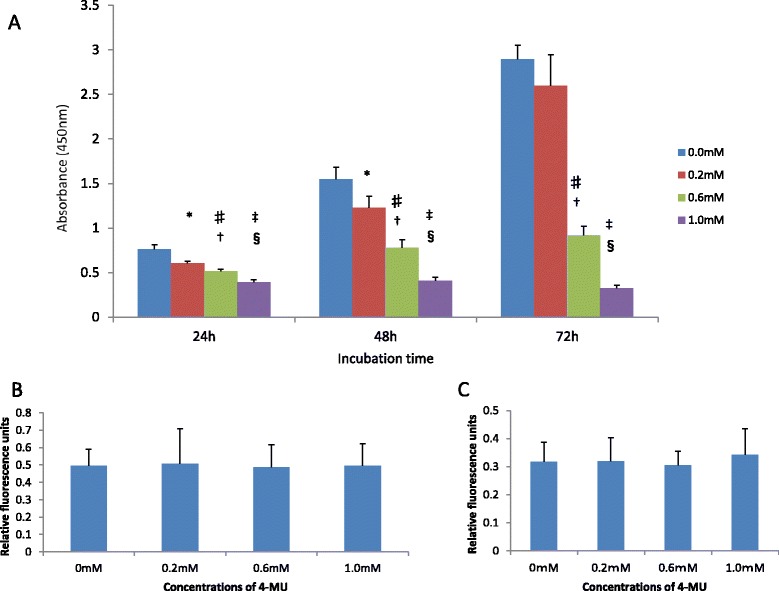
**Effects of 4-MU on cell proliferation, invasion, and migration. (A)** 4-MU significantly inhibited the proliferation of HRA cells in a dose-dependent manner. 4-MU did not affect cell invasion **(B)** and migration **(C)**. *P < 0.005 versus control, ♯*P* < 0.0001 versus control, ‡*P* = 0.0000 versus control, †*P* < 0.0001 versus 0.2 mM, § *P* = 0.0000 versus 0.2 mM, ¶*P* = 0.0000 versus 0.6 mM.

Figure 2A shows cell viability assessed by measuring A_450_ with a Multiskan FC microplate reader 3 hours after the addition of CCK-8. The cell viability of control cells had increased approximately 4-fold between 24 and 72 hours after plating, while proliferation of cells treated with 0.2, 0.6, or 1.0 mM 4-MU decreased over time, suggesting that 4-MU significantly inhibited the proliferation of HRA cells in a dose-dependent manner. However, 4-MU treatment did not affect cell invasion or migration (Figure 2B and C).

### Altered TP protein and mRNA expression levels in HRA cells treated with 4-MU

**Figure 3:**
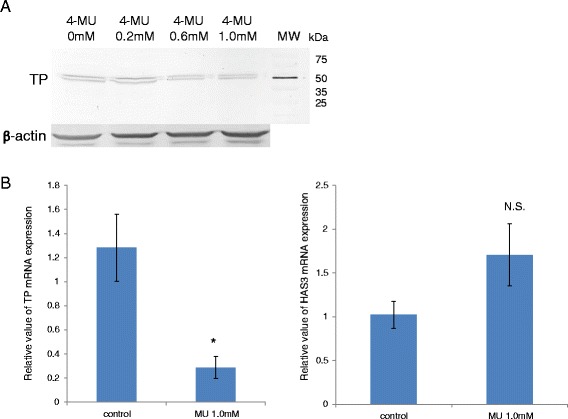
**4-MU treatment altered TP protein and mRNA expression levels in HRA cells. (A)** Western blot showed a reduction of TP protein in a dose-dependent manner of 4-MU. MW abbreviated molecular weight. **(B)** 4-MU administration significantly reduced the TP mRNA expression level in HRA cells (left panel), whereas there was no significant difference in HAS3 expression between HRA cells cultured with and without 4-MU (right panel). The relative expression of TP and HAS3 mRNA was analyzed by qRT-PCR in HRA cells cultured with or without 1.0 mM 4-MU for 24 hours. The data presented are the average ± SD of the relative mRNA expression values normalized to the 18S rRNA amount (*indicates *P* = 0.0005 compared with control).

Western blot analysis showed that 4-MU reduced TP protein level in a dose-dependent manner (Figure 3A). Real-time quantitative PCR confirmed a significant reduction of TP mRNA expression level in HRA cells treated with 1.0 mM 4-MU, whereas no significant difference in HAS3 expression was observed between HRA cells cultured with and without 4-MU (Figure 3B). Since HAS2, CD44, and VEGF endogenous mRNA expression levels were very low in HRA cells although their expression levels were high in U373MG human astrocytoma cells [18] and SH-SY5Y human neuroblastoma cells [20], it was impossible to evaluate the effect of 4-MU treatment (data not shown).

## Discussion

Survival was significantly prolonged by the administration of 4-MU to rats with peritonitis carcinomatosa resulting from inoculation with HRA cells. 4-MU also showed potential for reducing peritoneal dissemination of tumors as well as malignant ascites production. Rats were not administered 4-MU after day 14; however, the effect lasted until the end of the experiment. Although the rats that survived for 5 weeks had small peritoneal masses, there were no malignant ascites in their abdomen, suggesting that 4-MU treatment inhibits implantation and proliferation of malignant cells in these animals. Because 3 of the 4-MU-treated rats died of peritonitis carcinomatosa by day 13, technical aspects such as administration route and optimal dose of 4-MU must be reconsidered.

In the *in vitro* experiments, 4-MU inhibited proliferation of HRA cells in a dose-dependent manner, supporting previous results obtained in other types of cancer cells [13-15,21,22]. Although previous studies showed that 4-MU inhibited cancer growth by inhibiting cell invasion and migration [11,12,22], the present study did not show an effect of 4-MU on HRA cell invasion and migration. Although counting the number of cells that moved to lower membrane by staining with hematoxylin might be more appropriate in order to detect a little difference, this discrepancy may be partly accounted for by the absence of CD44 receptors on the surface of HRA cells. 4-MU is shown to elicit its action through inhibiting HAS that plays an important role in tumor growth via binding to CD44 receptors located on surface of cancer cells. Moreover, Day and Prestwich [23] suggested the existence of other receptors, indicating that 4-MU might exert its anti-proliferative effect directly or via an unknown receptor.

The antitumor mechanism of 4-MU remains poorly understood. HA, which is a high-molecular-weight glycosaminoglycan and a major component of extracellular matrix, promotes tumor proliferation, invasion, and migration by binding to the CD44 HA receptor [24]. HA is produced by HAS1, HAS2, and HAS3. Ovarian serous adenocarcinomas express HAS2 and HAS3 [25], and Anttila et al. [26] reported that high levels of stromal HA predict poor prognosis in patients with epithelial ovarian cancer. It is known that the HA-CD44 interaction can lead to the activation of intracellular signaling pathways such as PI3K/Akt that affect the proliferation, migration, and invasion of cancer cells [27]. Recently, CD44 was shown to be a cancer stem cell marker [10], and it was reported that CD44 could promote cell motility and tumorigenicity [11]. Earlier studies suggested that 4-MU exerted its antitumor activity by downregulating HAS and CD44 mRNA levels, leading to inhibition of HA synthesis as well as HA-CD44 binding [13]. However, this study revealed that HRA cells expressed extremely low levels of HAS2 and CD44 mRNA and that the expression level of HAS3 mRNA in HRA cells was low and was not altered by 4-MU administration, so HA production was not determined in this study. These results suggest that the anti-tumor effect of MU is dependent on a mechanism other than HA-CD44 signaling. Since hepatocellular carcinoma (HCC) generally arises in a cirrhotic liver, it is important to investigate HCC development in association with a fibrotic microenvironment [15]. 4-MU has anti-fibrogenic activity. Piccioni et al. [15] reported that 4-MU might be an anti-cancer agent for HCC associated with advanced fibrosis because it induced apoptosis of hepatic stellate cells (HSCs), which play a key role in advancing liver fibrosis, and decreased the number of activated HSCs. Saito et al. also reported that 4-MU inhibited cell proliferation and induced apoptosis in breast cancer cells together with inhibiting HA synthesis accompanied by downregulation of HAS2 mRNA levels in a dose-depending manner [28]. Therefore, 4-MU may have different anti-tumor mechanisms depending on the type of cancer.

It is well known that progression and metastasis of ovarian cancer are largely attributable to the activity of different angiogenic factors [29,30] and that the main route of development of this disease is via peritoneal dissemination with malignant ascites [19]. A recent study indicated that 4-MU inhibited angiogenesis *in vitro* and *in vivo* [31]. Thus, we determined the expression levels of VEGF and TP in HRA cells cultured with or without 4-MU. VEGF is a multifunctional cytokine and it is well known as an angiogenic factor [32]. Bevacizumab, a monoclonal antibody against VEGF, is used clinically as a treatment for ovarian cancer [3]. TP catalyzes the reversible phospholysis of thymidine, deoxyuridine, and their analogs to their respective bases and 2-deoxyribose-1-phosphate [33]. TP is identical to platelet-derived endothelial cell growth factor and has angiogenic factor activity [34]. Its expression in ovarian cancer patients is associated with a poor prognosis [35]. Tsukagoshi et al. [36] reported that angiogenesis in ovarian serous adenocarcinoma cell lines, including HRA, is dependent on TP mRNA expression and treatment with a TP inhibitor could block angiogenesis in those cell lines. Recently, some studies suggested that TP has an anti-apoptosis activity in addition to its angiogenic activity [37,38]. The present study showed that although HRA cells did not express VEGF mRNA, TP mRNA expression was significantly decreased by 4-MU treatment, suggesting that decreased TP expression may be involved in the inhibitory effect of 4-MU on ovarian cancer. Ko et al. [39] reported that PI3K/Akt signaling regulated TP expression. Therefore, 4-MU might inactivate PI3K/Akt signaling by suppressing a ligand-receptor interaction different from the HA-CD44 interaction.

To our knowledge, this is the first study to report an inhibitory effect of 4-MU on ovarian cancer. Although earlier studies showed that 4-MU exhibited its anti-tumor activity by suppressing the HA-CD44 interaction, this study demonstrated that 4-MU downregulated TP expression in ovarian cancer cells. This suggests that 4-MU may act as an angiogenesis inhibitor and have potential as a new drug in the therapeutic strategy for ovarian cancer.
